# SHP099-containing multi-targeting hydrogel promotes rapid skin reconstruction through modulating a variety of cells

**DOI:** 10.3389/fbioe.2025.1564827

**Published:** 2025-04-07

**Authors:** Zhixiao Liu, Lei Chen, Bingbing Hao, Yijin Hou, Chuan Lv, Yuanjie Zhu, Chaofeng Han

**Affiliations:** ^1^ Department of Histology and Embryology, Basic Medical College, Naval Medical University, Shanghai, China; ^2^ School of Health Science and Engineering, Shanghai Institute of Technology, Shanghai, China; ^3^ Department of Dermatology, Naval Medical Center, Naval Medical University, Shanghai, China; ^4^ Department of Plastic and Reconstructive Surgery, Shanghai Fourth People’s Hospital, School of Medicine, Tongji University, Shanghai, China; ^5^ Department of Histology and Embryology National Key Laboratory of Immunity and Inflammation, Naval Medical University, Shanghai, China

**Keywords:** multi-targetinghydrogel, wound healing, SHP099, macrophage, myofibroblast, hair follicle dermal shell

## Abstract

**Introduction:**

Adult wound scarring result in functional skin deficits. However, the development of effective measures to modulate the entire wound healing to encourage the skin function reconstruction is still a clinical challenge, as multiple cells are involved in wound healing hierarchically. Hydrogel scaffolds with long-lasting local release provide new insights into the clinical relevance of entire wound healing.

**Methods:**

Herein, a multi-targeting hydrogel loaded with SHP099 (Gel-SHP) is designed to modulate multiple cells during wound repair.

**Results:**

Our results show that Gel-SHP promotes rapid reconstruction of wound skin by modulating macrophages in the inflammatory stage, fibroblasts in the regeneration stage and smooth muscle cells in the remodelling stage. Gel-SHP could increase M2 macrophage differentiation and remodel the dermal shell of hair follicles through in situ release. Moreover, Gel-SHP may modulate myofibroblasts to promote wound contraction through SHP099-scaffold synergistic interactions.

**Discussion:**

Our results provide new insights into the design of functional hydrogels for tissue regeneration applications. Gel-SHP as a promising tool could provide new clues and new research paradigms for future studies and understanding of the wound healing process and dermal shell formation.

## 1 Introduction

Wound healing is a complex process involving multiple tissues and multiple cell lineage interactions (Gurtner et al., 2008). The macrophage-dominated inflammatory stage occurs approximately 0–3 days after wound formation by recruiting peripheral inflammatory cells infiltration. Macrophages remove damaged tissue in preparation for tissue regeneration. The fibroblast-dominated regeneration stage occurs approximately 3–10 days after wound formation. The differentiation and activation of fibroblast in dermis and basal stem cell in epidermis will induce regeneration. Fibroblasts close the wound by cell proliferation, remodeling collagen, and promoting angiogenesis. The remodelling stage is initiated after the wound closes, starts around day 10 and lasts for several months. The involvement of smooth muscle cells in the reconstruction of skin appendages during the remodelling stage restores the skin’s sensory and thermoregulatory functions (Martin, 1997; Gurtner et al., 2008; Aragona et al., 2017; Talbott et al., 2022). However, in the absence of effective clinical interventions for the entire process of wound healing, adult wound repair often ends with the formation of scars with a disorganized accumulation of collagen and fibroblasts. Lost skin appendages affect skin function. Therefore, adult wound repair has been a clinical challenge. Hydrogels as scaffolds can release drugs *in situ* for long periods and loaded with drugs have the potential to modulate the entire process of wound healing in clinical therapy. Functional hydrogels loaded drugs have become a new hotspot in scar treatment research (Li and Mooney, 2016; Shan and Wu, 2023), which has been shown a great potential for application as an ideal drug delivery system in promoting wound healing and regeneration of skin appendages (Liang et al., 2021; Wang et al., 2023; Goh et al., 2024). However, whether functional hydrogels have the potential to modulate the entire wound healing process remains unclear to date.

**FIGURE 1:**
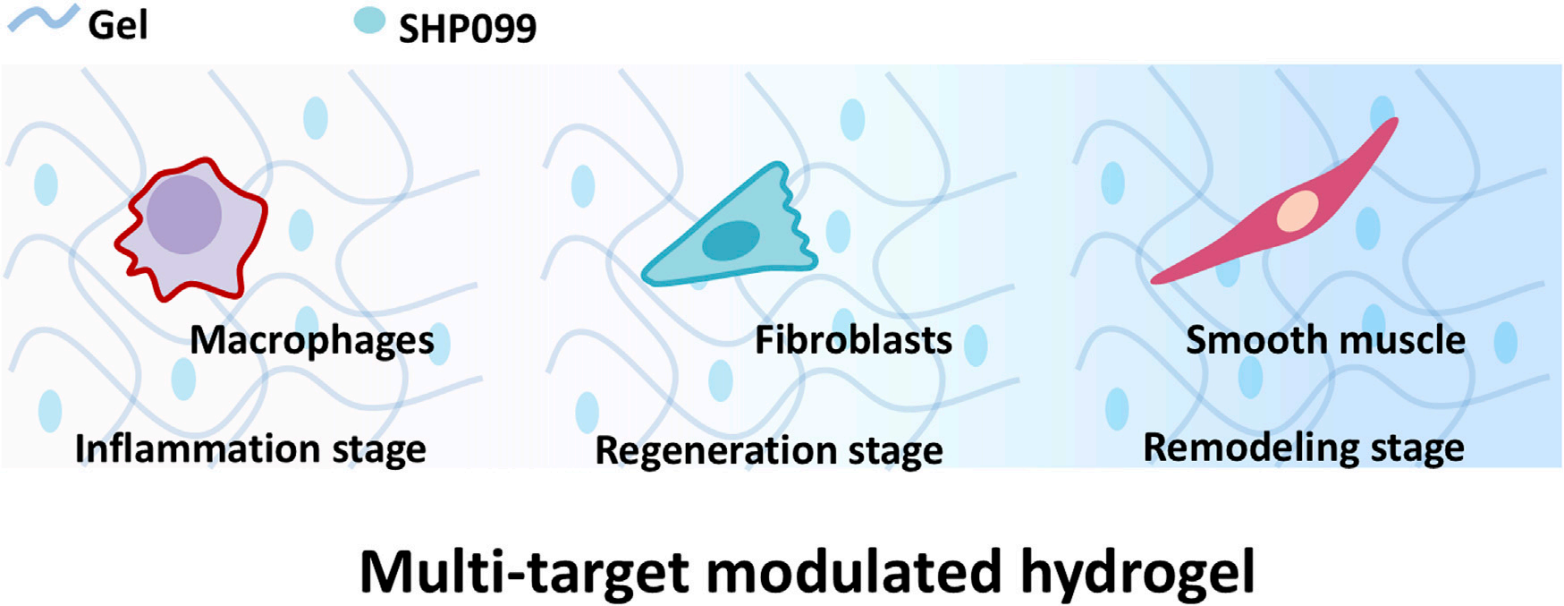
Gel loaded with SHP099 (SHP) to prepare multi-targeting hydrogel. The multi-targeting hydrogel is hypothesized to promote the reconstruction of wound skin structures by regulating macrophages, fibroblasts and smooth muscle cells at different stages of wound repair.

Hence, a multi-targeting functional hydrogel was designed to modulate the whole process and promote skin regeneration of wound repair to promote skin reconstruction. SHP2 as an important tyrosine phosphatase is involved in the regulation of multiple wound repair-associated cells (Östman et al., 2006). It has been shown that Src homology 2 domain containing protein tyrosine phosphatase (SHP2) regulates macrophage inflammatory responses (Sun et al., 2022; Ye et al., 2023; Ying et al., 2024), fibroblast function (Qin et al., 2012) and smooth muscle cell behavior (Rafiq et al., 2006; Marin et al., 2008; Burmeister et al., 2012) by phosphorating the tyrosine of both receptor and non-receptor tyrosine kinases. As a result, SHP2 inhibitor SHP099(Chen et al., 2016) was chosen as a multi-targeting drug to be loaded into a hydrogel scaffold Gelatin Methacryloyl (Gel, Gel) suitable for wound repair (Zhu et al., 2022) to prepare multi-targeting functional hydrogels (Gel-SHP). We hypothesize that multi-targeting hydrogels promote rapid wound recovery and correctly establish skin appendages to inhibit scar formation by inhibiting inflammation during the inflammatory stage, regulating fibroblasts during the regeneration stage and modulating smooth muscle during the remodelling stage (Figure 1).

Herein, Gel-SHP was used to treat full-thickness incision in mice. The data show that Gel-SHP has a modulatory effect on the whole process of wound repair. Gel-SHP promotes faster wound entry into the regeneration stage by inhibiting macrophage inflammatory response during the inflammatory stage. Then, Gel-SHP promotes wound closure during the regeneration stage by inducing myofibroblast differentiation via enhancing alpha Smooth Muscle Actin (alpha-SMA) expression. Ultimately, Gel-SHP contributes reconstruction of the hair follicle during the remodelling stage by encouraging the regeneration of the smooth muscle composed dermal shell through Caldesmon-1 (Cald1) expression promotion. Our work provides new ideas for functional hydrogel design and new clues for understanding the process of wound repair.

## 2 Materials and methods

### 2.1 Preparation of Gel and Gel-SHP

Gel and Gel-SHP were prepared as described in previous work (Li et al., 2020). To prepare Gel, 0.1 g lyophilised GelMA (EFL-GM-90, EFL-GelMA, China) was dissolved in 1 ml PBS containing 0.25% w/v Lithium phenyl (2,4,6-trimethylbenzoyl) phosphinate (LAP, EFL-GelMA, China), and the Gel solution was exposed to Ultraviolet (UV) for 30 s to form Gel hydrogel. To prepare Gel -SHP, 0.1 g lyophilised GelMA (EFL-GM-90, EFL-GelMA, China) was dissolved in 1 ml PBS containing 0.25% w/v LAP (EFL-GelMA, China) and 10 µM SHP099 (S0775, Selleck, USA). Finally the Gel-SHP solution was exposed to UV for 30 s to form Gel-SHP hydrogel.

### 2.2 Parameters of Gel and Gel-SHP

#### 2.2.1 Scanning electron microscope (SEM)

The prepared Gel and Gel-SHP were frozen at −80°C overnight and then transferred to a vacuum freeze dryer for 24 h. The ultrastructure of the obtained freeze-dried hydrogel samples was characterized using scanning electron microscopy (Hitachi U8010, Japan) after 45 s of gold spraying (Leica EM ACE200, Germany).

#### 2.2.2 Swelling ratio

Gel and Gel-SHP were prepared at 37°C and were weighed (W_a_). Next, Gel and Gel-SHP were incubated in equal volumes of PBS at 37°C and weighed (W_b_) after 24 h (Yuan et al., 2016). The swelling Ratio of Gel and Gel-SHP were calculated using the following equation:

$$\text{Swelling Ratio }\left(\%\right)=\frac{W_{b}-W_{a}}{W_{a}}\times 100\%$$ (1)

### 2.3 Gel-SHP drug release in vitro

The *in vitro* release of SHP099 from the Gel-SHP was measured according to previous description (Liu et al., 2018). One Gram Gel-SHP incubated in 10 ml Phosphate Buffered Saline (PBS) at 37°C and shaken at 100 rpm/min. One mL of release medium was aspirated on days 1, 2, 3, 4, 5 and 6 respectively, while 1 ml of PBS was added in the release medium. SHP099 content in release medium was assessed by measuring absorbance at 260 nm using UV spectrophotometer (PerkinElmer Lambda 25, USA). The release medium obtained from the same treatment of Gel was used as a blank control. The cumulative release of SHP099 was calculated using the following equation:

$$\text{Cumulative release }\left(\%\right)=\frac{ɑC_{n}+b\sum C_{n-1}}{W_{0}}\times 100\%$$ (2)

Where ɑ is total volume of PBS (10 ml), b is volume of PBS aspirated per measurement (1 ml), W_0_ is the SHP099 weight in Gel-SHP, C_n_ and C_n-1_ are the concentrations of SHP099 in release medium on day n and day n-1 respectively.

### 2.4 Animal experimentations

#### 2.4.1 Animal model of wound

Six-week-old male C57 mice from the Animal Centre of the Naval Military Medical University were anesthetized by intraperitoneal injection of 2% sodium pentobarbital (50 mg/kg). After shaving, a circular full-thickness incision of 7 mm in diameter was made in the back of the mouse by biopsy punch. The mice were then divided into two groups randomly as follows: 1) Gel group: the 30 μL Gel solution was added to the wound. Next the wound was exposed to UV for 30 s to form Gel hydrogel *in situ*. 2) Gel-SHP group: 30 μL Gel-SHP solution was added to the wound. Next the wound was exposed to UV for 30 s to form Gel-SHP hydrogel *in situ*. Then, the Tegaderm transparent film was used to cover the traumatized area to prevent mice from licking the wound and causing experimental errors. All the animal experimental procedures were supported by the Committee on Ethics of Biomedicine, Naval Medical University (Approval No: SYXK2022-0011).

#### 2.4.2 Wound healing rates

Photographs were taken, and mouse wounds were measured on days 0, 3 and 7 of recovery. The wound healing rate was calculated using the following equation:

$$\text{Wound healing rate }\left(\%\right)=\frac{\left(S_{0}-S_{t}\right)}{S_{0}}\times 100\%$$ (3)

Where S_0_ is the area of the wound on day 0 after surgery and S_t_ is the area of the wound on day t after surgery.

### 2.5 Cell culture and experiments

#### 2.5.1 Cell line

The mycoplasma-negative mouse macrophage cell line RAW264.7 and mouse fibroblast cell line L-929 were cultured in Dulbecco’s Modified Eagle Medium (DMEM, D5796, Sigma) supplemented with10% v/v fetal bovine serum (FBS, 10091148, Gibco, Grand Island, NY, USA), 100U/ml penicillin, and 100 µg/ml streptomycin (10378016, Gibco). All cells were cultured at 5% CO_2_ and 37°C.

#### 2.5.2 Macrophage-induced differentiation

Lipopolysaccharide (LPS, 100 ng/ml, SMB00610, Sigma) was added to the medium to induce macrophage differentiation in the direction of M1 for 12 h. The cells were lysed by Trizol (15596026, Invitrogen) for Real-Time Quantitative Reverse Transcription PCR.

#### 2.5.3 SHP099 macrophage stimulation experiment

SHP099 was added to the medium (5 μM) at the same time as LPS was added to induce macrophage differentiation towards M_1_ for 12 h. Then the cells were lysed by TRIzol (15596026, Invitrogen) for Real-Time Quantitative Reverse Transcription PCR.

#### 2.5.4 SHP099 fibroblast stimulation experiment

SHP099 was added to the medium (5 μM) to stimulate fibroblasts for 12 or 72 h. Then cells were lysed by RIPA buffer (89900, Thermo Scientific) supplemented with protease and phosphatase inhibitors (A32959, Thermo Scientific) for Western blot.

### 2.6 Real-time quantitative reverse transcription PCR

The total Ribonucleic Acid (RNA) obtained using TRIzol was reverse-transcribed for complementary Deoxyribonucleic Acid (cDNA) by cDNA reverse transcription kit (4374967, Applied Biosystems). Then, the expression level of IL-6 was assessed by QuantStudio 3 (Applied Biosystems) using SYBR Green R kit (11762500, Invitrogen). Primer sequence: GAPDH: forward primer: 5′-ACA​ACT​TTG​GTA​TCG​TGG​AAG​G-3′, reverse primer:5′- GCC​ATC​ACG​CCA​CAG​TTT​C-3′. IL-6: forward primer: 5′-TAG​TCC​TCC​CTA​CCC​CAA​TTT​CC-3′, reverse primer: 5′-TTG​GTC​CTT​AGC​CAC​TCC​TTC-3′ (Jimenez Calvente et al., 2020; Liu et al., 2022). Data were obtained using ΔΔCt method to obtain gene expression (GAPDH as an internal reference) using the Applied Biosystems qPCR software.

### 2.7 Hematoxylin and eosin staining (H&E staining)

A total of 18 six-week-old male C57 mice were randomly divided into two groups (Gel group and Gel-SHP group) and sampled on days 3, 7, and 14 (n = 3), respectively for HE staining and wound healing rate calculation. The 1ⅹ1 cm^2^ area of mouse skin tissue was taken around the wound at postoperative days 3, 7 and 14 respectively. Then tissues were fixed with 4% paraformaldehyde and paraffin sections were prepared longitudinally in the direction of the head and tail. The sections were stained with hematoxylin-eosin for histological characterization.

#### 2.7.1 Epithelial thickness

Epidermal thickness above the wound was measured in wounds that had healed over 7 days by epidermal cell morphology characterization.

#### 2.7.2 Hair follicle analysis

The density of hair follicles in regenerating skin was counted. The skin region 1 mm length next to the wound was defined as regenerated skin because the wound shrank from 7 to 5 mm in diameter on day 3 after surgery and to 3 mm by day 7 after surgery.

### 2.8 Immunohistochemical staining

A total of 10 six-week-old male C57 mice were randomly divided into two groups (Gel group and Gel-SHP group) and sampled on day 7 for Immunohistochemical staining, Immunofluorescence Staining and Masson staining. A 1 × 1 cm^2^ area of mouse skin tissue was taken around the wound at postoperative day 7.Mouse skin tissues sections were deparaffinized and incubated with CD206 primary antibody (24595, CST) overnight at 4°C. The sections were then incubated with HRP-labelled secondary antibodies (SA100001-2, Proteintech). After reaction with the substrate (8114, CST), CD206 positive cells were stained brown. Mouse skin tissues sections were deparaffinised and incubated with Caldesmon-1 (Cald1) primary antibody (12503, CST) or alpha Smooth Muscle Actin (alpha-SMA) primary antibody (19245, CST) overnight at 4°C. The sections were then incubated with AP-labelled secondary antibodies (18653, CST). After reaction with the substrate (76713, CST), Cald1 or alpha-SMA positive cells were stained red. All immunohistochemical images were analysed using ImageJ and IOD/Area were performed to obtain the AOD of the positive areas.

### 2.9 Masson staining

Mouse skin tissue sections were deparaffinized and staining with Weigert’s iron hematoxylin. The sections were then stained using Ponceau S and Aniline blue, respectively. Collagen fibres were stained blue and cellular tissue was stained red. All Masson staining images were analysed using ImageJ and IOD/Area were performed to obtain the AOD of the positive areas.

### 2.10 Transcriptome RNA sequencing (RNA-Seq)

A total of 6 six-week-old male C57 mice were randomly divided into two groups (Gel group and Gel-SHP group) and sampled on day 7 for Transcriptome RNA Sequencing (RNA-Seq). Mouse wound tissue was obtained on day 7 after surgery. Total RNA was collected by the TIANSeq mRNA Capture Kit (NR105, TianGen). Mouse wound tissue transcriptomic data were obtained via the Illumina platform. Data in which *p* value < 0.05 and log_2_(Fold change) > 2 were defined as significantly different genes.

#### 2.10.1 Data analysis

Significantly different genes were analyzed based on previous article descriptions (Liu et al., 2024). Significantly different genes with FDR<0.05 were selected for Gene Ontology (GO) or Kyoto Encyclopedia of Genes and Genomes (KEGG) enrichment analysis. Significant enrichment was defined as *p* value < 0.05. The top five with the smallest p-value in GO biological progress (BP) were listed to reveal the biological function of the significantly different genes. The top thirty with the smallest p-value in KEGG were listed to explain the potential regulatory mechanisms of significantly different genes.

### 2.11 Immunofluorescence staining

#### 2.11.1 Slices

Mouse skin tissue sections were deparaffinized and incubated with Ki67 primary antibody (9129, CST) overnight at 4°C. The sections were then incubated with Alexa Fluor®Plus 488-labelled secondary antibodies (A32731, Invitrogen). Nuclei were labelled with DAPI (62248, Invitrogen). After fluorescent labelling the sections were imaged using a fluorescence microscope (DM2500, Leica).

#### 2.11.2 Cultured cells

The cultured L929 cells were fixed using 4% paraformaldehyde and then incubated with Cald1 primary antibody overnight at 4°C. The cells were then incubated with Alexa Fluor™ 633-labelled secondary antibodies (A-21070, Invitrogen). Nuclei were labelled with DAPI (62248, Invitrogen). After fluorescent labelling the cells were imaged using a laser confocal microscopy (LSM980, Zeiss).

### 2.12 Western blot

Cultured L929 cells were lysed by RIPA and the proteins were separated using SDS-PAGE. Then the proteins were transferred to polyvinylidene fluoride (PVDF) membranes (IPVH00010; Millipore, Burlington, MA, USA) by wet transfer. The PVDF membranes were then incubated with Cald1 primary antibody or alpha-SMA primary antibody or GAPDH primary antibody overnight at 4°C. The PVDF membranes were then incubated with HRP-labelled secondary antibodies (SA100001-2, Proteintech). After reaction with the substrate in an enhanced chemiluminescence kit (SQ202, Epizyme), the protein signals were collected using a chemi-luminescence system (ChemiScope; Clinx Science).

### 2.13 Statistical analysis

All data were analyzed using t-tests in Origin 2024 software (OriginLab). For cell fluorescence and tissue slide stained (H&E, Immunohistochemical and Immunofluorescence) images, Image-Pro Plus 6.0 (Media Cybernetics) was used for quantitative photometric analysis. All gene expression sequencing data acquisition and data analyses were obtained by Tiangen Company.

## 3 Results

### 3.1 Gel hydrogel with multi-targeting inhibitor SHP099 (Gel-SHP) promotes rapid reconstruction of wound skin

**FIGURE 2:**
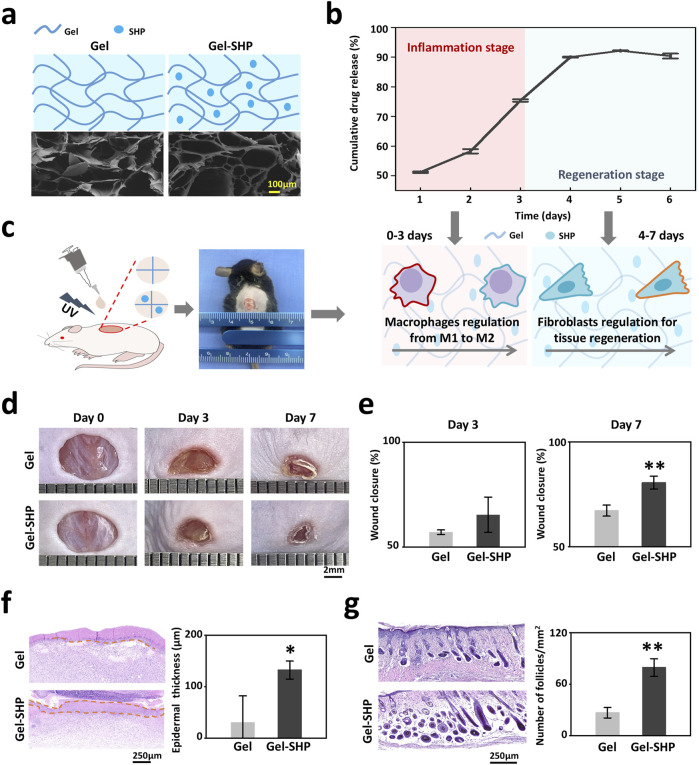
Gel-SHP promotes wound healing and skin remodeling. **(a)** Scanning electron microscopy (SEM) morphology characterization of Gel hydrogels (Gel) and Gel hydrogels loaded with the multi-targeting inhibitor SHP099 (Gel-SHP). **(b)** Cumulative release profile of SHP in Gel-SHP (n = 3). We hypothesized that Gel-SHP would regulate macrophages during the inflammatory stage and fibroblasts during the tissue regeneration stage. **(c)** Mouse wound model. **(d, e)** Gel-SHP promotes wound closure. **(d)** Photographs of wounds treated by Gel and Gel-SHP for day 0, 3 and 7 (1 mm per grid). **(e)** The wound closure rate between groups Gel and Gel-SHP was not significant on day 3 (n = 3, *p* = 0.15427), whereas the wound closure rate of Gel-SHP group was significantly higher than Gel group on day 7 (n = 3, *p* = 0.00453). **(f)** H&E-stained sections (left, day 7 of treatment) show the epidermal thickness (brown dash-dotted line) was significantly higher in group Gel-SHP than in group Gel (right, arrows, n = 3, *p* = 0.03237). **(g)** H&E-stained sections on day 14 treatment show (left) hair follicle number in Gel-SHP group was significantly higher than in Gel group (right, n = 3, *p* = p = 0.00158). *p < 0.05; **p < 0.01; ***p < 0.001; ****p < 0.0001.

Since SHP2 has the ability to phosphorylate a wide range of tyrosine kinases to regulate a multitude of cellular behaviors (Feng, 2007),the multi-target SHP2 inhibitor (SHP099) was loaded into UV-induced light-cured Gel to prepare Gel-SHP099 hydrogels (Gel-SHP, 10 μM SHP099). Characterization of Gel and Gel-SHP showed that SHP099 did not significantly alter the microscopic morphology (Figure 2a) and swelling ratio of Gel (Supplementary Figure S1) (Equation 1). The cumulative release rate of SHP099 in Gel-SHP was then characterized (Figure 2b) (Equation 2). Mouse wound model was used to evaluate the effects and mechanisms of Gel-SHP on wound healing (Figure 2c). The dorsal skin of 6-week C57 male mice was excised in full thickness (7 mm in diameter). Then Gel or Gel-SHP was cured on the wound by light induction for treatment. Wound closure rate was assessed on days 3 and 7 (Equation 3). Sections of mouse wound tissue were prepared for H&E staining on day 7 and 14. There was no significant difference between Gel and Gel-SHP wound closure rates on day 3 (Figures 2d, e), which may suggest that the Gel-SHP has no effect on wound closure during the early inflammatory stage. In contrast, results showed that Gel-SHP significantly accelerated wound closure (Figures 2d, e) and promoted wound epidermal regeneration (Supplementary Figure S2 red box; Figure 2f) on day 7, and significantly formed more hair follicles on day 14 (Supplementary Figure S3; Figure 2g) compared to Gel. The above data indicates that Gel-SHP can significantly promote wound closure in the wound regeneration stage and promote hair follicle regeneration in the wound remodelling stage.

### 3.2 Gel-SHP promotes macrophage in wound differentiation towards M2 during inflammatory stage

**FIGURE 3:**
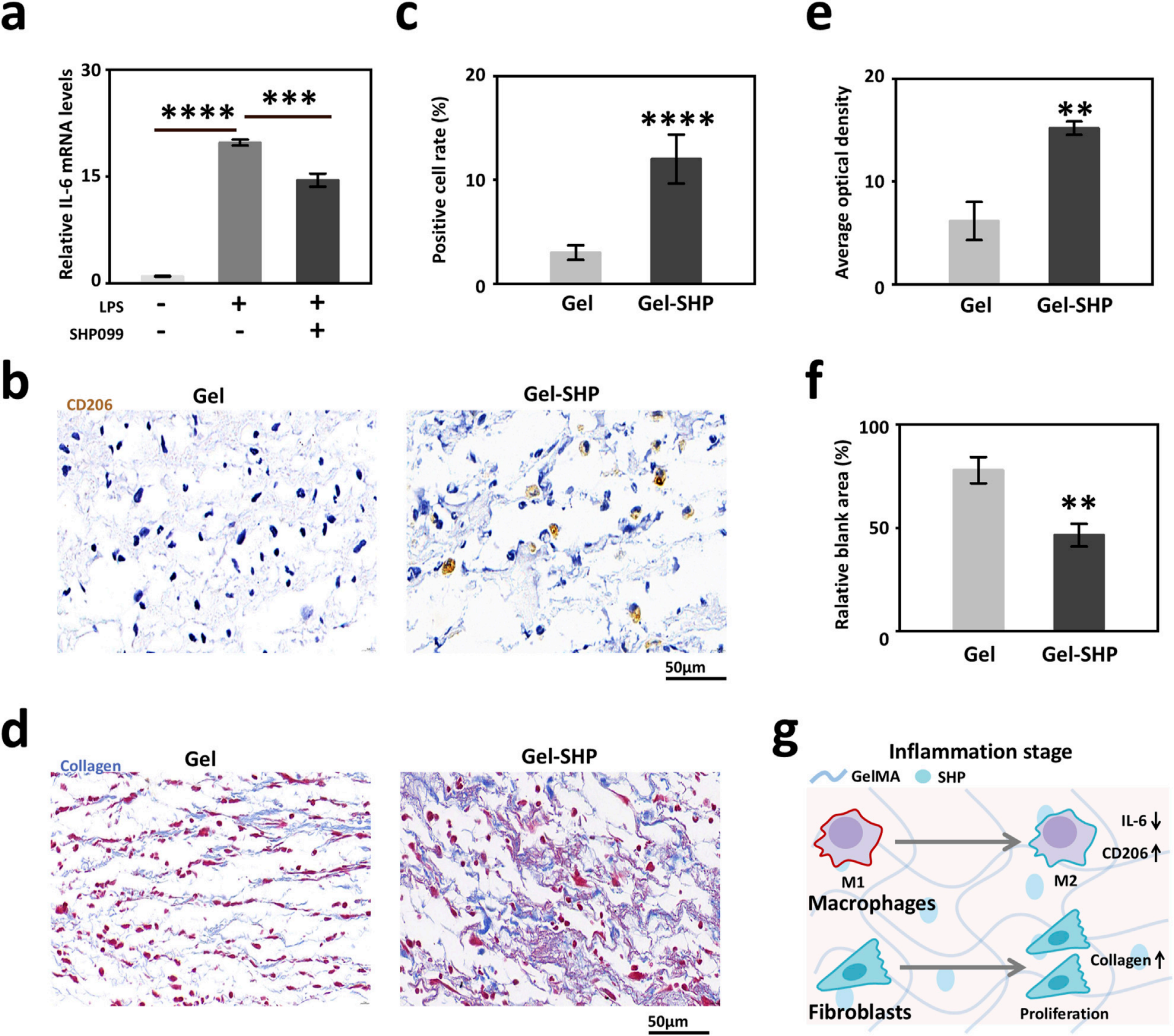
Gel-SHP regulates macrophages during inflammatory stage and promotes tissue regeneration. **(a)** Q-PCR results of IL-6 expression in macrophages (RAW264.7; LPS-/SHP099- VS LPS+/SHP099-, n = 3, *p* = 1.71445E-7; LPS+/SHP099- VS LPS+/SHP099+, n = 3, *p* = 8.43562E-4). **(b, c)** Gel-SHP promotes M2 differentiation of macrophages in wounds. **(b)** Immunohistochemically (IHC) stained sections (day 3 of treatment; CD206, brown). **(c)** The percentage of macrophages positive for CD206 expression in Gel-SHP group was higher than in the Gel group (n = 5, *p* = 3.60348E-5). **(d–f)** Gel-SHP promotes collagen formation and fibroblast regeneration in the wound. **(d)** Masson stained sections (day 3 of treatment; collagen, blue; fibroblast, red) **(e)** The blue average optical density (AOD) representing collagen was significantly higher in Gel-SHP group than in Gel group (n = 5, *p* = 0.00135). **(f)** The blank area was significantly smaller in Gel-SHP group than in Gel group (n = 5, *p* = 0.00291). **(g)** Gel-SHP regulates the differentiation of wound macrophages from M1 to M2, while promoting fibroblast proliferation and facilitating collagen synthesis during the inflammatory stage. *p < 0.05; **p < 0.01; ***p < 0.001; ****p < 0.0001.

To clarify whether Gel-SHP inhibits the wound inflammatory response during the inflammatory stage (days 0–3), the modulatory effects of SHP099 on mouse macrophages was analyzed. LPS (100 ng/ml) was added to mouse macrophage cell (RAW264.7) medium for 12 h to induce macrophage differentiation into the M1 phenotype (Kawai et al., 1999; Orihuela et al., 2015). SHP099 (5 μM) was also added to the medium to determine whether SHP099 inhibited inflammatory factor secretion in M1 macrophages. IL-6 gene expression was used to assess the level of macrophage inflammatory factor secretion (Orihuela et al., 2015). The results showed that SHP099 significantly reduced macrophage IL-6 expression (Figure 3a). Further, the macrophage M2 phenotype in mouse wounds on day 3 was characterized by immunohistochemical (IHC) staining for CD206 in the wound sections (Cho et al., 2014; Tang et al., 2019). The results showed that the percentage of CD206 positive cells at the wound was significantly higher in the Gel-SHP group than in the Gel group (Supplementary Figure S4; Figures 3b, c). The above results suggest that Gel-SHP promotes the differentiation of macrophages in the wound towards the M2 phenotype. Previous work has shown that the presence of macrophage M2 is an important signal for the initiation of tissue regeneration in wounds (Talbott et al., 2022), so the collagen production and fibroblastic regeneration characteristics were further analysed by day 3 wounds tissue Masson staining sections (Zhu et al., 2022). The results showed that the amount of collagen synthesis in the Gel-SHP group wound was significantly higher than that in the Gel group (Supplementary Figure S5; Figures 3d, e), and the blank area of tissue in the Gel-SHP group wound was significantly lower than that in the Gel group (Supplementary Figure S5; Figures 3d, f), and a lower percentage of blank area means more tissue reconstruction at the wound. The above results suggest that Gel-SHP can promote the shortening of the inflammatory stage and induce tissue regeneration by modulating the differentiation of traumatic macrophages towards the M2 phenotype (Figure 3g).

### 3.3 Gel-SHP promotes wound healing by facilitating alpha smooth muscle actin (alpha-SMA) expression and angiogenesis in wound tissue during regeneration stage

**FIGURE 4:**
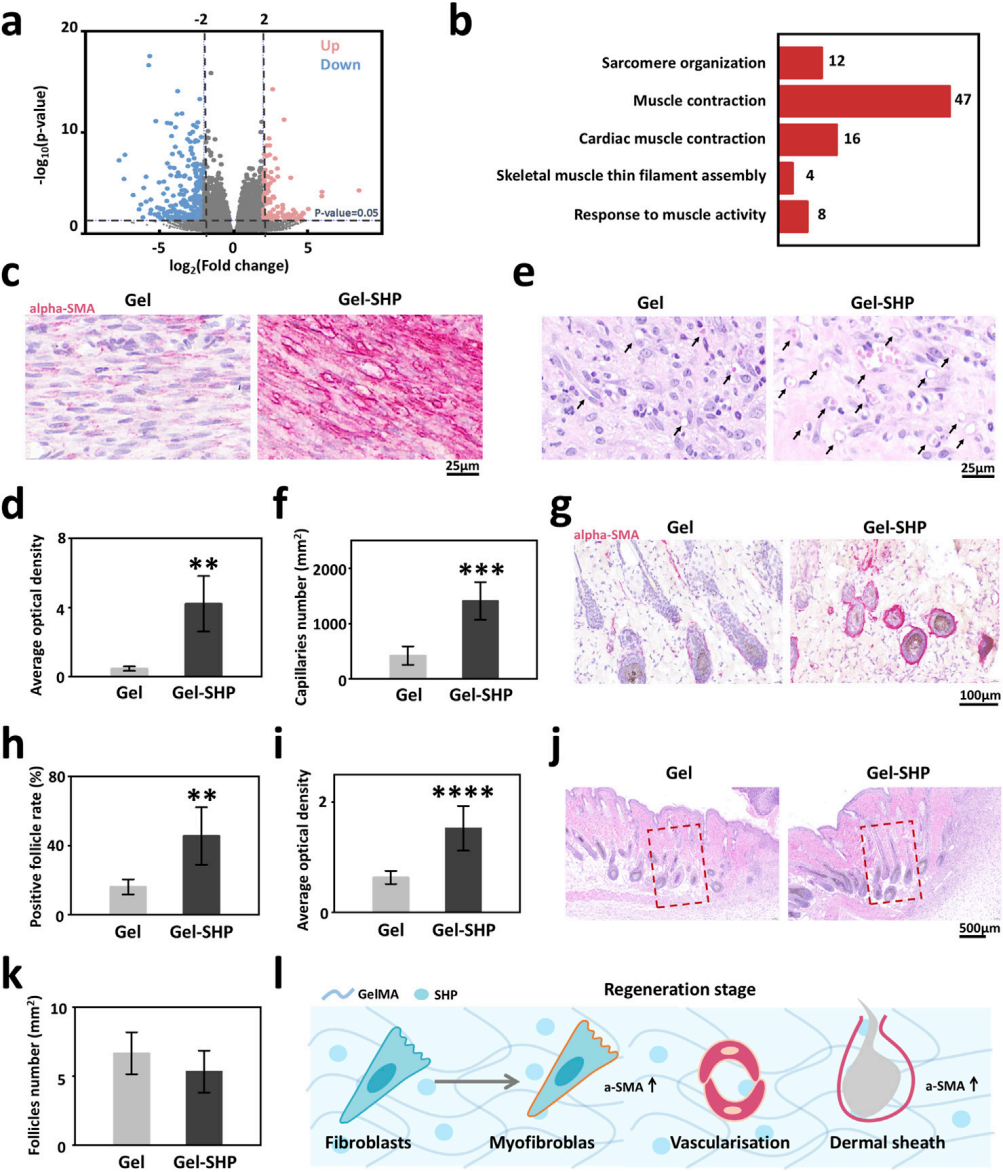
Gel-SHP regulates fibroblasts during regeneration stage and promotes angiogenesis. **(a, b)** Transcriptomic sequencing reveals Gel-SHP regulated genes and gene functions in the regeneration stage. **(a)** Volcano map of differentially expressed genes (day 7 of treatment; Gel-SHP vs Gel, n = 3). P < 0.05, log_2_ (Fold change) > 2 was defined as significant difference. **(b)** Gene Ontology (GO) enrichment analysis of significantly upregulated genes in biological progress (BP). Significant enrichment was defined as *p* value < 0.05 (Top 5 in BP). **(c, d)** Gel-SHP promotes fibroblast alpha-smooth muscle actin (alpha-SMA) expression in wounds. **(c)** IHC stained sections of alpha-SMA (day 7 of treatment; red). **(d)** The AOD of alpha-SMA in fibroblasts of Gel-SHP group is significantly higher than Gel group (n = 5, *p* = 0.00125). **(e, f)** Gel-SHP promotes angiogenesis of the wound. **(e)** H&E-stained sections (7 day of treatment). **(f)** Statistical results show that the number of blood vessels at the Gel-SHP group wound was significantly higher than that in Gel group (n = 5, *p* = 3.8429E-4). **(g–i)** Gel-SHP promotes alpha-SMA expression in neo-regenerated hair follicles. **(g)** IHC stained sections of alpha-SMA (day 7 of treatment; red). **(h)** The alpha-SMA positive hair follicle rate was significantly higher in Gel-SHP group than Gel group (n = 5, *p* = 0.00499). **(i)** The hair follicle alpha-SMA AOD in Gel-SHP group was significantly higher than that Gel group (n = 5, *p* = 2.88749E-6). **(j, k)** Gel-SHP did not significantly increase the number of hair follicles after day 7 of treatment. **(j)** H&E-stained sections (day 7 of treatment). **(k)** The difference in the number of hair follicles regenerated in the wound (Regeneration area: red box = 1 mm^2^) between Gel-SHP group and Gel group was not significant (n = 3, *p* = 0.34). **(l)** Gel-SHP modulates the differentiation of fibroblasts into myofibroblasts, promotes angiogenesis and promotes alpha-SMA expression during the regeneration stage. *p < 0.05; **p < 0.01; ***p < 0.001; ****p < 0.0001.

To explore the mechanism of how Gel-SHP promotes wound closure, wound tissues from mice on day 7 were collected for transcriptome sequencing and data analysis. In total of the 36,405 genes measured, the Gel-SHP group showed a significant increase in the expression of 133 genes and a significant decrease in the expression of 297 genes in the wounds compared with the Gel group (Supplementary Table S1; Figure 4a). To further analyses the biological mechanisms by which differentially expressed genes promote wound closure, significantly differentially expressed genes were subjected to Gene Ontology (GO) enrichment analysis, and biological progress (BP) in GO enrichment analysis allowed identification of the biological processes that the differentially expressed genes mainly focused on regulating (Liu et al., 2024). The results showed significant downregulation of genes mainly enriched in inflammation-related biological processes in the Gel-SHP group compared to the Gel group (Supplementary Table S2; Supplementary Figure S6). This suggests that Gel-SHP can still significantly inhibit inflammation during the regeneration stage. Significantly upregulated genes in the Gel-SHP group were mainly enriched in biological processes related to muscle tissue (Supplementary Table S2; Figure 4b). It has been shown that myofibroblasts with muscle tissue characteristics play a key role in wound closure (Tomasek et al., 2002; Younesi et al., 2024). Therefore, we hypothesized that Gel-SHP may promote wound closure by promoting myofibroblast formation. The alpha-Smooth Muscle Actin (alpha-SMA) was used to characterize myofibroblasts at the wound (Wynn and Ramalingam, 2012). The results showed that alpha-SMA expression was significantly higher at the wound in the Gel-SHP group compared with the Gel group (Supplementary Figure S7 black box; Figures 4c, d). The above results indicated that Gel-SHP promoted myofibroblast formation at the wound during regeneration stage. A large number of circular cavity morphologies with high alpha-SMA expression appeared in the Gel-SHP group in immunohistochemically (IHC) stained sections (Figure 4c). We therefore hypothesized that Gel-SHP may promote angiogenesis in the wound. Consequently, the number of blood vessels at the wound on day 7 was counted in H&E stained sections, based on the typical histological morphology of the vessels. The results showed that the number of vessels at the wound was significantly higher in Gel-SHP group than Gel group (Supplementary Figure S2 blue box; Supplementary Figure S9; Figures 4e, f). The above results suggest that Gel-SHP promotes angiogenesis during regeneration stage. An interesting phenomenon was revealed in the immunohistochemically stained wound sections, where alpha-SMA expression around neonatal hair follicles (hair follicles within 2 mm next to the wound were defined as neonatal hair follicles) was significantly higher in the Gel-SHP group than in the Gel group (Supplementary Figure S7 blue box; Figures 4g–i). The above results indicate that Gel-SHP promotes myofibroblast formation at the wound to facilitate wound closure by significantly increasing the expression of alpha-SMA. Meanwhile Gel-SHP significantly promoted wound angiogenesis during regeneration stage. Gel-SHP also promoted elevated alpha-SMA expression around hair follicles in the vicinity of the wound, but did not significantly promote an increase in the number of neoplastic hair follicles during regeneration stage (Supplementary Figure S2 black box; Figures 4j–l). Surprisingly, SHP099 did not significantly promote alpha-SMA expression in the fibroblast cell line L929 *in vitro* experiments (Supplementary Figure S8).

### 3.4 Gel-SHP promotes hair follicle regeneration by elevating Caldesmon-1 (Cald1) expression

**FIGURE 5:**
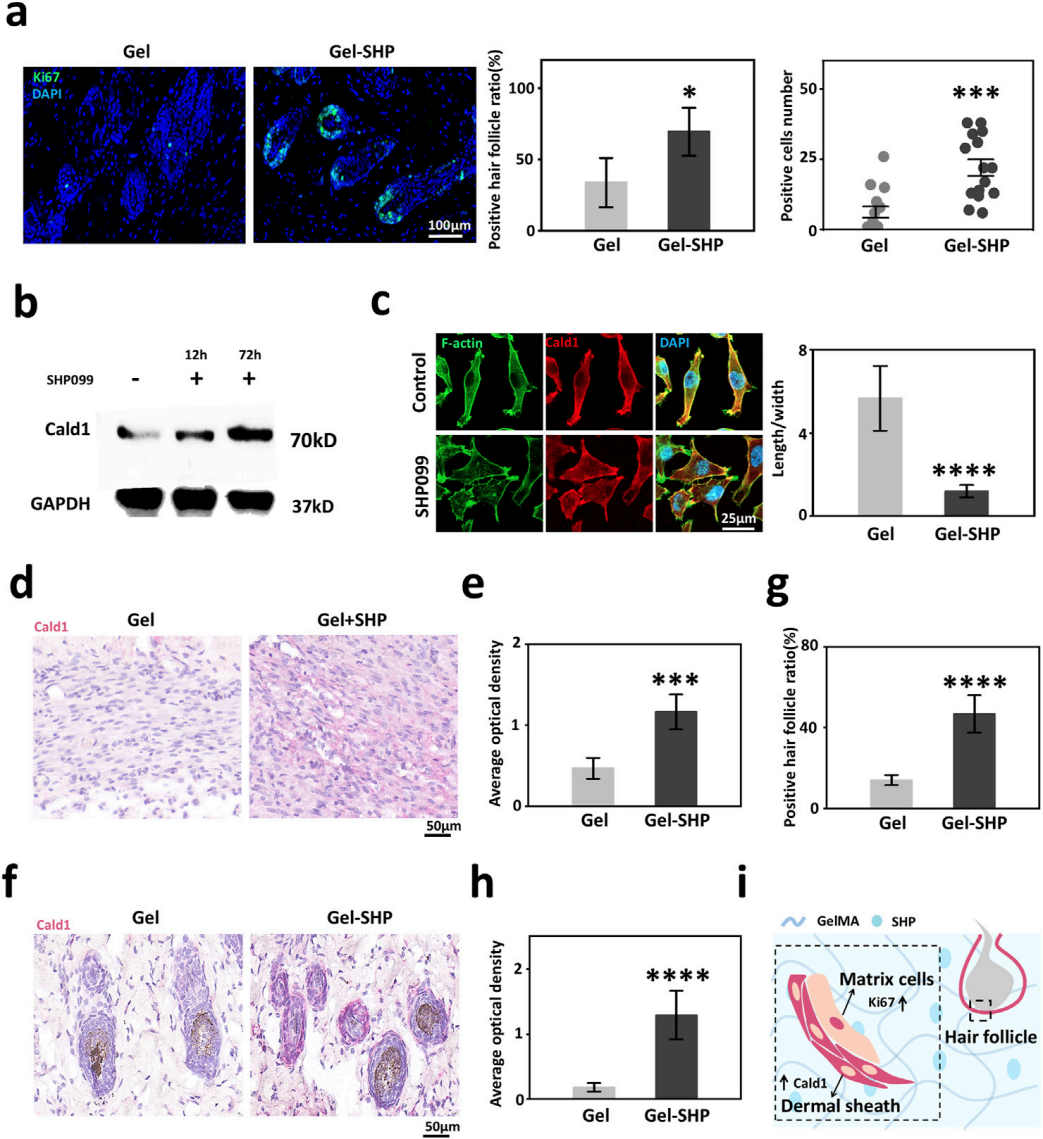
Gel-SHP promotes hair follicle regeneration via modulating Cald1 expression during the remodeling stage. **(a)** Gel-SHP promotes Ki67 expression in neonatal hair follicle matrix cells. Immunofluorescence (IF) stained sections (left; 7 day of treatment). The Ki67-positive follicles (follicles with Ki67-positive cells were defined as positive follicles) ratio (n = 5, *p* = 0.01081) and the number of positive cells in positive follicles (15 randomly selected hair follicles:3 follicles close to the wound per section, n = 15, *p* = 1.058E-4) were significantly higher in Gel-SHP group than in Gel group. **(b)** Western blot results of L929 showed that SHP099 promoted Cald1 expression in fibroblasts. **(c)** IF results (left, day 3 incubated) showed that SHP099 altered the morphology of fibroblasts and significantly decreased the length/width ratio of fibroblasts (n = 10, *p* = 1.17055E-9). **(d, e)** Gel-SHP promotes Cald1 expression in wound fibroblasts. **(d)** IHC stained sections of Cald1 (day7 of treatment; red). **(e)** Cald1 AOD in wound fibroblasts in Gel-SHP group was significantly higher than that in Gel group (n = 5, *p* = 2.62218E-4). **(f–h)** Gel-SHP promotes Cald1 expression in neo-regenerated hair follicles. **(f)** IHC stained sections of Cald1 (day 7 of treatment; red). **(g)** Cald1 positive neo-regenerated hair follicle percentage in Gel-SHP group was significantly higher than that in Gel group (n = 5, *p* = 6.04142E-5). **(h)** Cald1 AOD in neo-regenerated hair follicles in Gel-SHP group was significantly higher than that in Gel group (10 randomly selected hair follicles:2 follicles close to the wound per section, n = 10, *p* = 2.86295E-8). **(i)** Gel-SHP maintains the dermal shell of hair follicles to promote follicular regeneration by promoting Cald1 expression during the remodeling stage. *p < 0.05; **p < 0.01; ***p < 0.001; ****p < 0.0001.

High expression of alpha-SMA in hair follicles in the vicinity of the wound implies that Gel-SHP may lead to an increase in hair follicles by maintaining the dermal shell of the hair follicle. It has been shown that alpha-SMA high-expressing dermal shell is critical for the maintenance of proliferation of hair follicle stem cell-associated matrix cells (Jahoda and Reynolds, 2001; Fuchs, 2007; Heitman et al., 2020), so we hypothesized that Gel-SHP could promote the proliferative capacity of neonatal hair follicle matrix cells. Ki67 was used to characterized the proliferative capacity of hair follicle matrix cells (Fuchs, 2007). The results showed that the number of Ki67-positive hair follicles and the number of Ki67-positive cells in hair follicles were significantly higher in the Gel-SHP group compared to the Gel group (Supplementary Figure S10; Figure 5a). To analyses the mechanism by which Gel-SHP promotes dermal shell formation, mouse day 7 skin tissue transcriptome data were further analyzed by Kyoto Encyclopedia of Genes and Genomes (KEGG) enrichment analysis to look for Gel-SHP-regulated signaling pathways. Upregulated genes in the Gel-SHP group were enriched for the calcium signaling pathway (Supplementary Table S3; Supplementary Figure S11). Therefore, we hypothesized that Caldesmon-1 (Cald1) related to calcium signaling pathway (Kordowska et al., 2006), which plays a key role in dermal shell formation (Heitman et al., 2020), may be regulated by Gel-SHP. Indeed, the results show that SHP099 significantly upregulated Cald1 expression in fibroblast cell line L929 *in vitro* experiments (Figure 5b; Supplementary Figure S12). Immunofluorescence results showed that SHP099 significantly remodeled L929 cell morphology (Figure 5c). Further, both wound Cald1 expression (Supplementary Figure S13 black box; Figures 5d, e) and peri-neonatal hair follicle Cald1 expression (Supplementary Figure S13 red box; Figures 5f–h) were higher in the Gel-SHP group than Gel group. The above results suggest that Gel-SHP can promote the formation of the dermal shell of hair follicles by promoting Cald1 expression and may promote hair follicle regeneration by maintaining the dermal shell around the nascent hair follicle in remodelling stage (Figure 5i).

## 4 Discussion and conclusion

In previous studies we have demonstrated that Gel hydrogels are effective in promoting wound repair (Zhu et al., 2022), and here we discuss the modulatory effects of the functional Gel hydrogel loaded with SHP099 on a variety of cells in the wound healing process, using Gel as a control group (Qiao et al., 2019). The inflammatory response that is rapidly initiated after tissue injury plays a key role in tissue reconstruction. Pro-inflammatory M1-type macrophages are recruited to the wound and remove dead cells and infectious organisms during the inflammatory stage. Subsequently, pro-repair M2 macrophages help in tissue reconstruction during the regeneration stage. Eventually, as the wound appendage structure is rebuilt, the immune cells are eliminated through apoptosis or leave the injury site during the remodelling stage. Acute or chronic inflammation mediated by M1-type macrophages present in both regeneration stage and remodelling stage will abnormally activate fibroblasts, leading to fibrosis and eventual scar formation (Eming et al., 2017). Scar formation leads to impaired skin function in adult wound healing. Functional hydrogels provide a new means of clinical treatment of scars. SHP2 inhibitors have been shown extensively to regulate macrophages, fibroblasts and smooth muscle cells (Sun et al., 2022; Ye et al., 2023; Ying et al., 2024; Qin et al., 2012; Rafiq et al., 2006; Marin et al., 2008; Burmeister et al., 2012). Therefore, we designed Gel-SHP, a multi-target functional hydrogel that promotes rapid reconstruction of skin structure by modulating the master cells in the three stages of wound repair. In the inflammatory stage, Gel-SHP promotes the wound advancement into the regenerative stage through the ability of SHP099 to inhibit the macrophage inflammatory response and modulate macrophage switching towards M2. In the regenerative stage, Gel-SHP accelerates wound closure by promoting angiogenesis and myofibroblast differentiation via enhancing the alpha-SMA expression in a low inflammatory response microenvironment. Interestingly, in established studies, high expression of alpha-SMA in wound tissue tends to imply fibrosis (Teng et al., 2022), however, our results show that myofibroblast with high expression of alpha-SMA could promote wound closure. The above results imply that alpha-SMA may have a complex function in the wound healing process, and the use of Gel-SHP system may provide us with clues to discover new mechanisms of wound healing. Small vessel regeneration is essential for rapid wound healing. Existing studies have focused on the process of vascular epithelial cell differentiation, and little is known about the role of other cells such as smooth muscle cells, and perivascular myofibroblasts in the process of vascular regeneration. Vascular endothelial stem cells in tissues undergo the process of endovascular progenitors (EVP, CD31^low^ VEGFR2^−/low^) and transient amplifying (TA, CD31^int^VEGFR2^−/low^), to become definitive differentiated (D, CD31^high^VEGFR2^high^) cells (Chambers et al., 2021). However, until today we know little about the regulation of EVP, TA and D by myofibroblasts. In the future, we will use the Gel-SHP system to analyze the regulation of vascular endothelial stem cells by myofibroblasts, to clarify the mechanism of vascular regeneration promoted by Gels-SHP. Gel-SHP promotes and maintains the dermal shell of the hair follicle by facilitating the expression of Cald1, which promotes the reconstruction of the wound skin appendages in the remodelling stage by modulating the behaviors of smooth muscle cell. Dermal sheaths play a key role in hair follicle regeneration and regulation of the hair follicle cycle (Martino et al., 2023). However, little is known about the mechanisms of dermal sheath formation and maintenance (Martino et al., 2020). So far we know little about the mechanism of follicular shell formation, and the lack of small molecule compounds that promote follicular shell formation is the main reason. Our results show that SHP099 significantly promotes the expression of genes related to follicular shell differentiation in fibroblasts and promotes follicular shell regeneration. The above results suggest that SHP099, as an ideal small molecule drug, will provide important clues for the future understanding of hair follicle shell formation. Finally, the Gel-SHP is expected to provide an ideal 3D culture system for studying hair follicle regeneration. Although several methods have been used to prepare controlled drug release hydrogel scaffolds, the complexity of the interactions between the tissue microenvironment and the hydrogel scaffolds has led to the fact that until now we know little about the in-situ drug release behavior of hydrogel scaffolds *in vivo*. This has led us to discuss SHP099 regulation of fibroblasts using cell lines that do not mimic the regulatory effects of Gel-SHP on fibroblasts *in vivo*. Subsequently, we will look for methods that can accurately characterize the release behavior of hydro coagulation scaffolds *in vivo* to further clarify the mechanism by which Gel-SHP promotes wound repair.

In summary, multi-target functional Gel-SHP that can be used to modulate the entire process of wound healing have been designed and applied to the treatment of wounds. Our results show that the Gel-SHP promotes macrophage differentiation towards M2 for wound regeneration during the inflammatory stage. Furthermore, Gel-SHP promoted myofibroblast formation to facilitate wound closure in a low-inflammatory response environment in the regeneration stage. Crosstalk between hydrogel and SHP099 may play a key role in this process. Moreover, Gel-SHP promotes the regeneration of the hair follicles dermal shell by up-regulating Cald expression during the remodelling stage. Our results provide new insight into the design of functional hydrogels for wound repair. Meanwhile, our work provides new clues for understanding the wound repair process and hair follicle regeneration.

## Data Availability

The original contributions presented in the study are included in the article/Supplementary Material, further inquiries can be directed to the corresponding authors.
