# Supplementary material for: SHP099-containing multi-targeting hydrogel promotes rapid skin reconstruction through modulating a variety of cells
Source: Front Bioeng Biotechnol. 2025 Apr 7;13:1564827. doi: 10.3389/fbioe.2025.1564827 (PMC12009829; doi:10.3389/fbioe.2025.1564827)
Supplement: Supplementary file 1 [file DataSheet1.zip › Supplementary_Material-2/Supplementary_Material.docx]

Supplementary Material

Supporting Information


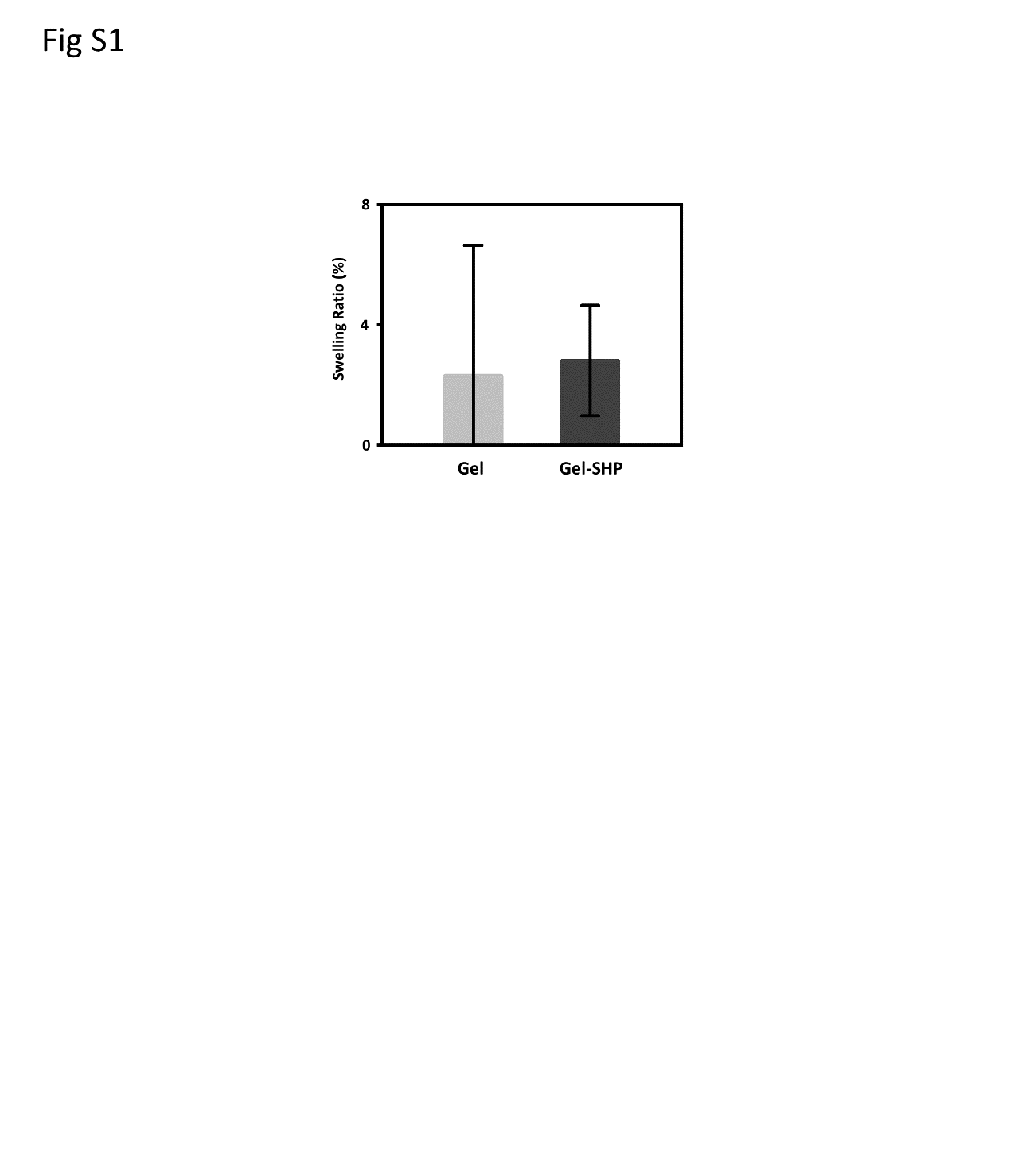


Figure S1 Swelling Ratio of Gel and Gel +SHP


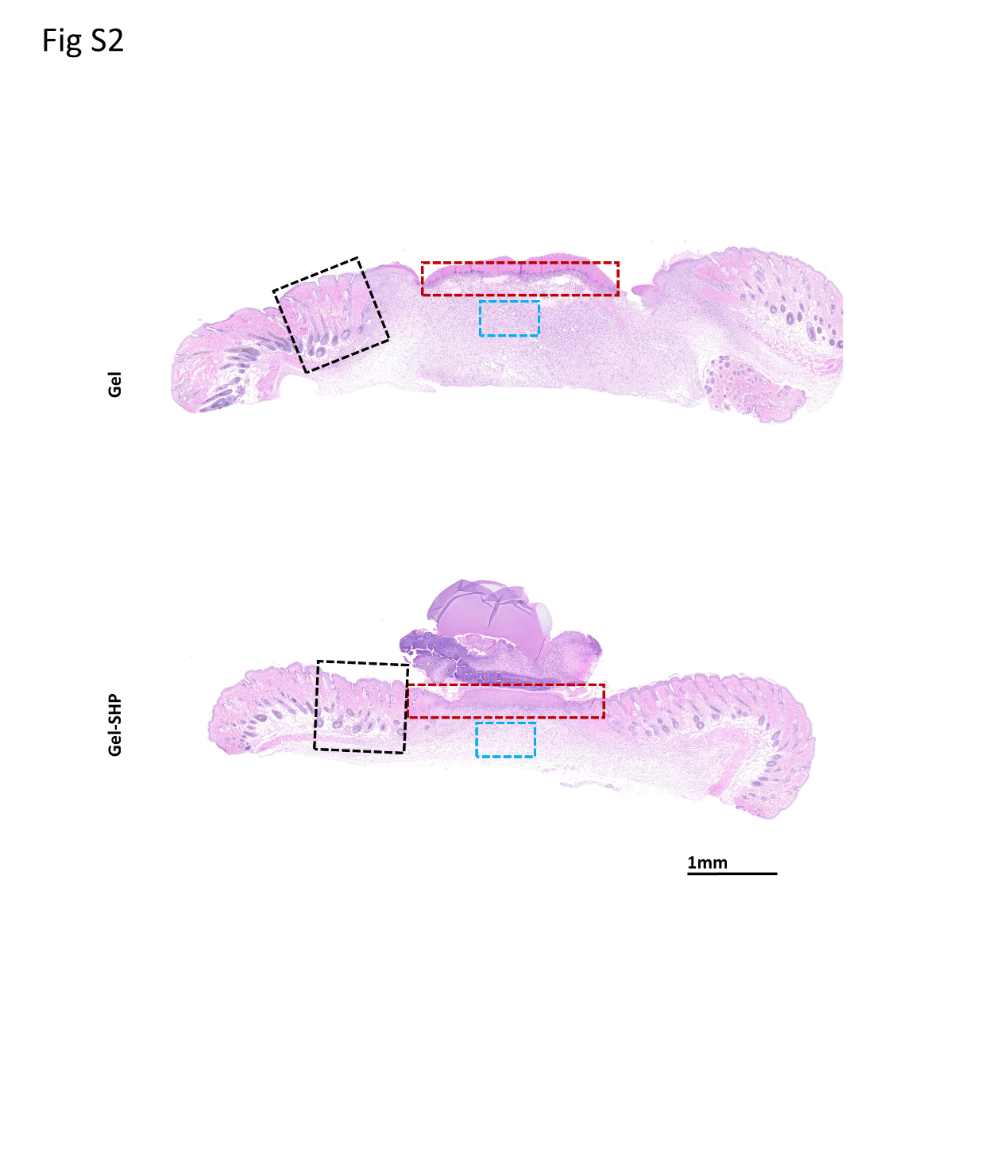


Figure S2 Section stained with H&E after 7 days of Gel and Gel-SHP treatment of wounds.


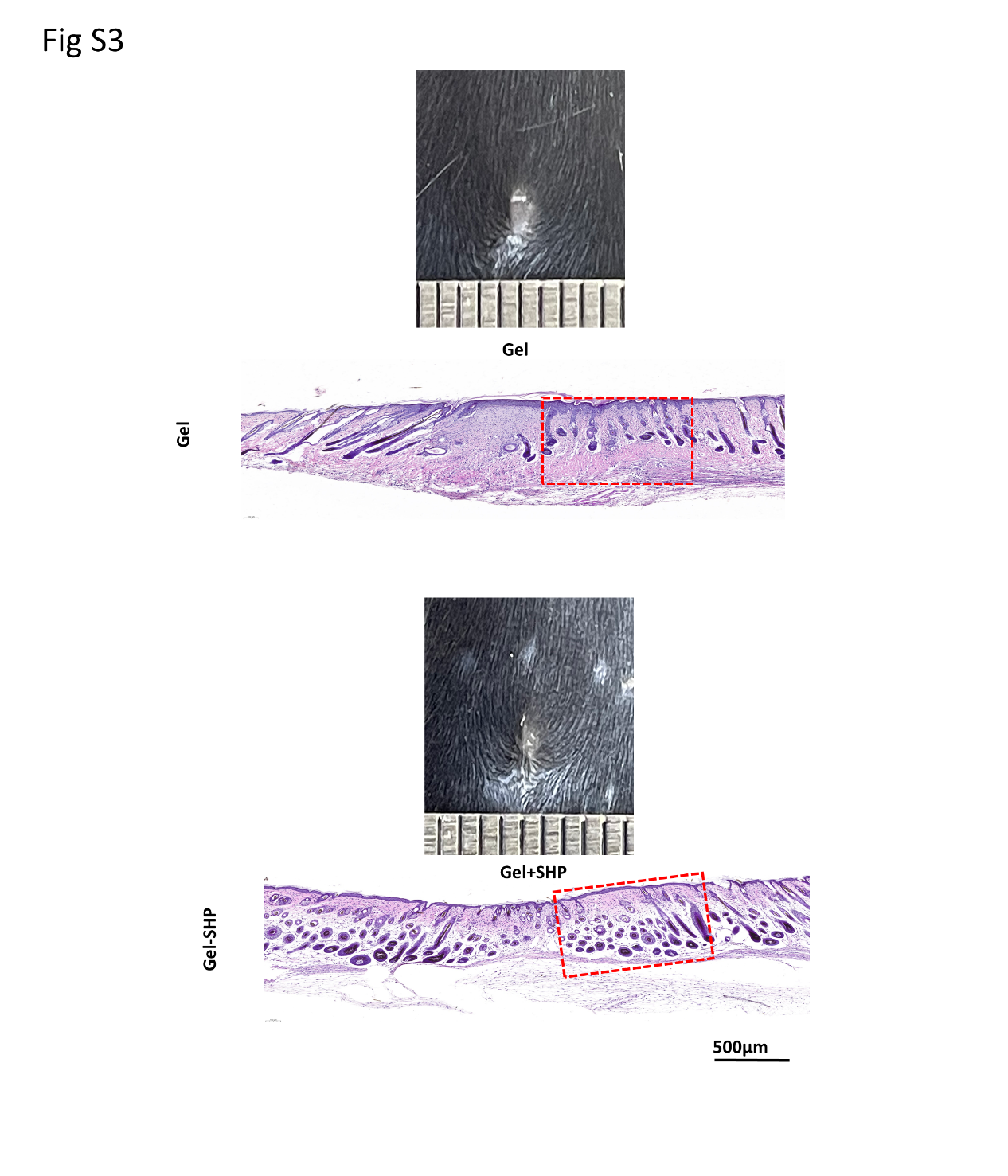


Figure S3 H&E stained sections after 14 days of Gel and Gel-SHP treatment of wounds.


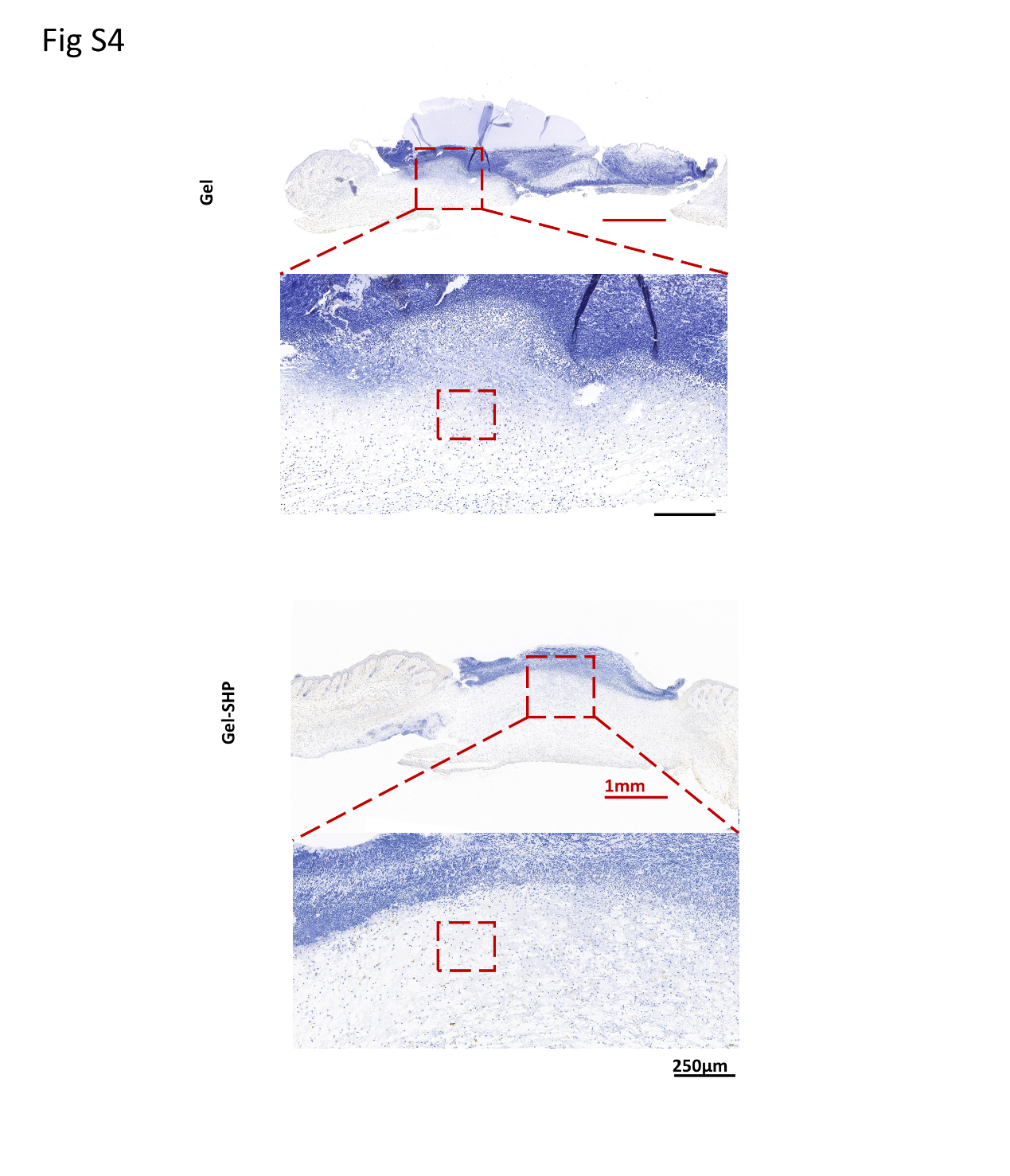


Figure S4 Immunohistochemically (IHC) stained sections after 3 days of Gel and Gel-SHP treatment of wounds (CD206, brown).


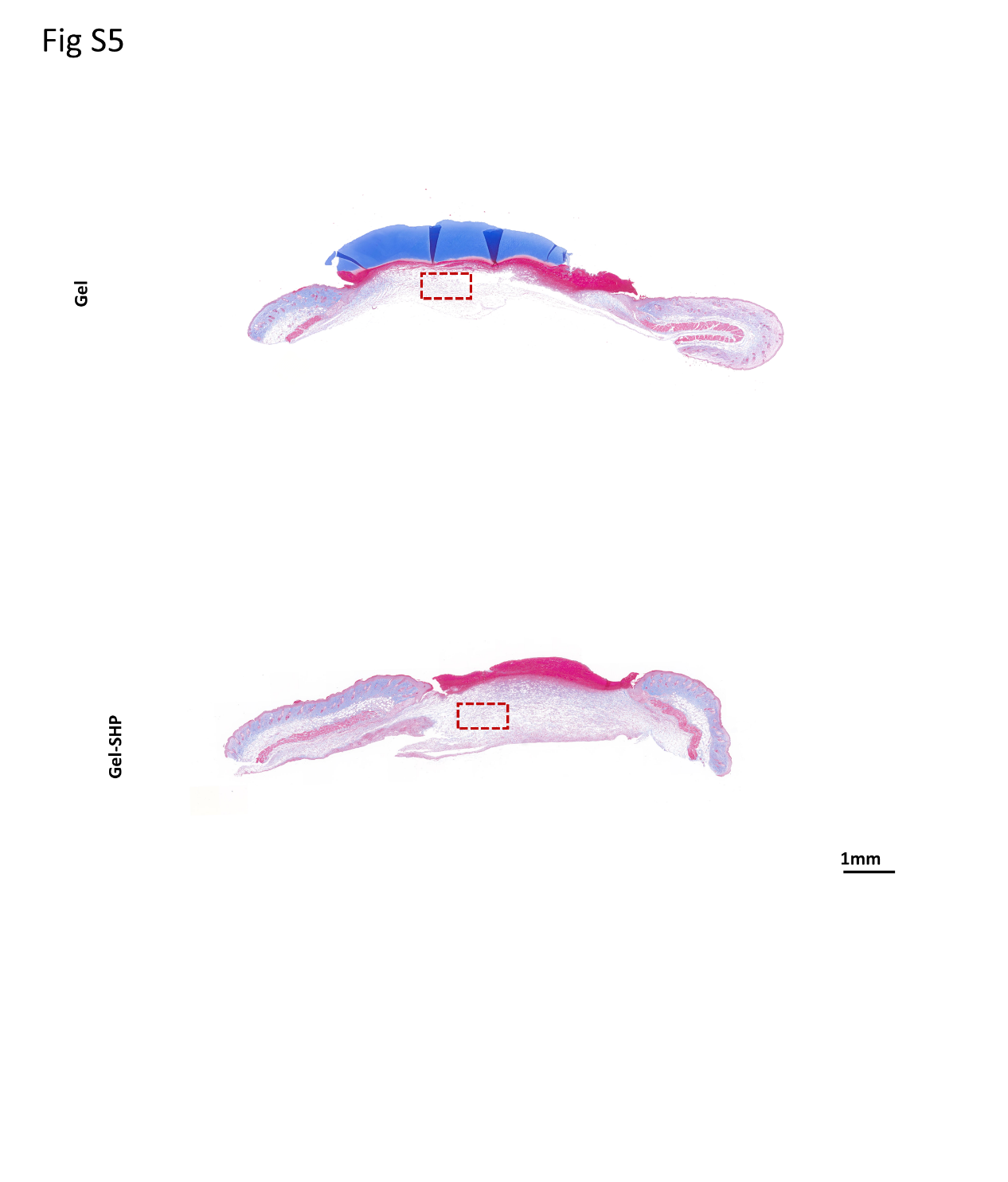


Figure S5 Masson stained sections after 3 days of Gel and Gel-SHP treatment of wounds.


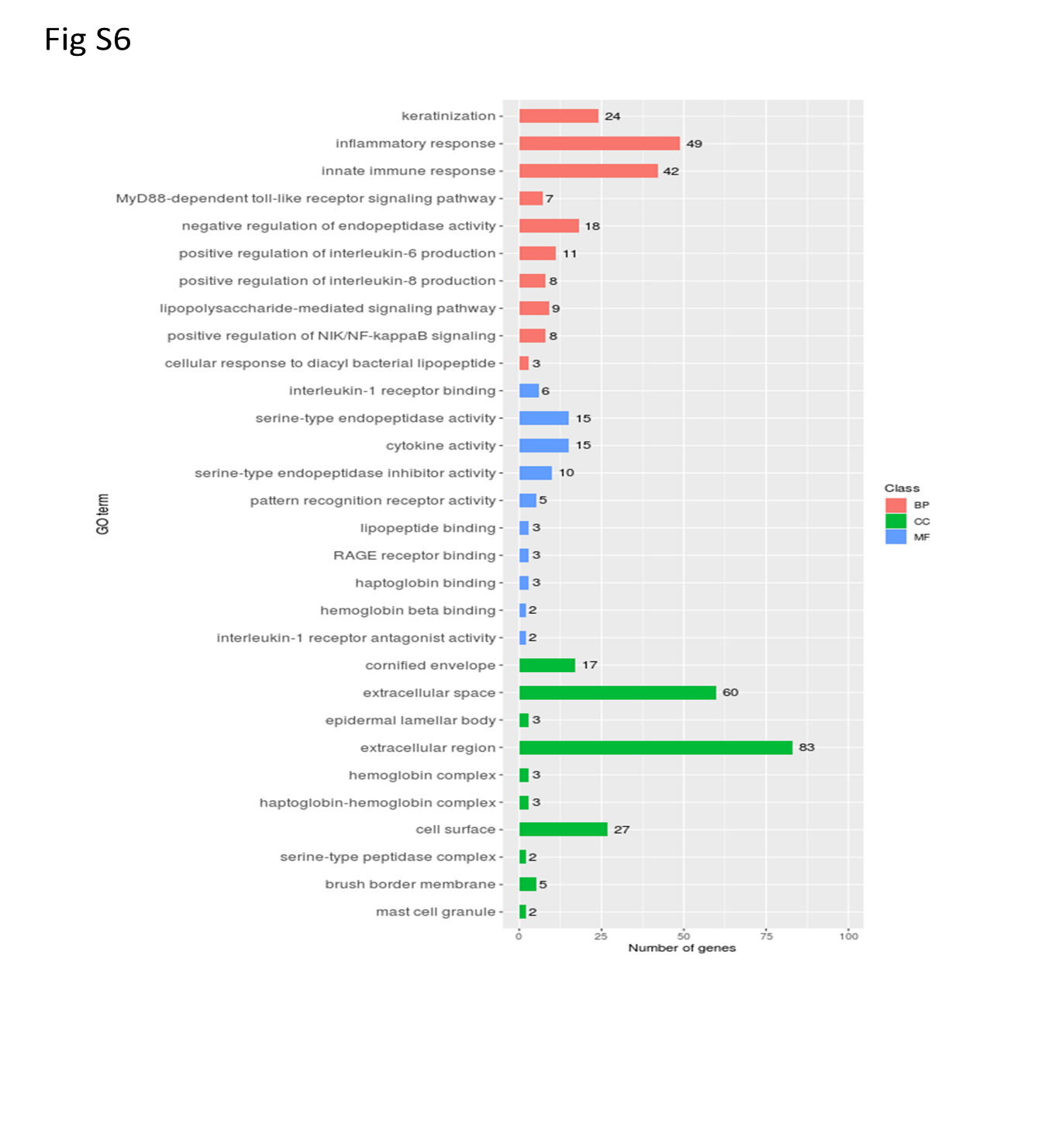


Figure S6 Gene Ontology (GO) enrichment analysis of significantly down-regulated genes in biological progress (BP), Cellular Component (CC) and Molecular Function (MF). Significant enrichment was defined as *p* value<0.05 (Gel-SHP group VS Gel group, Top 5 in BP, CC and MF).


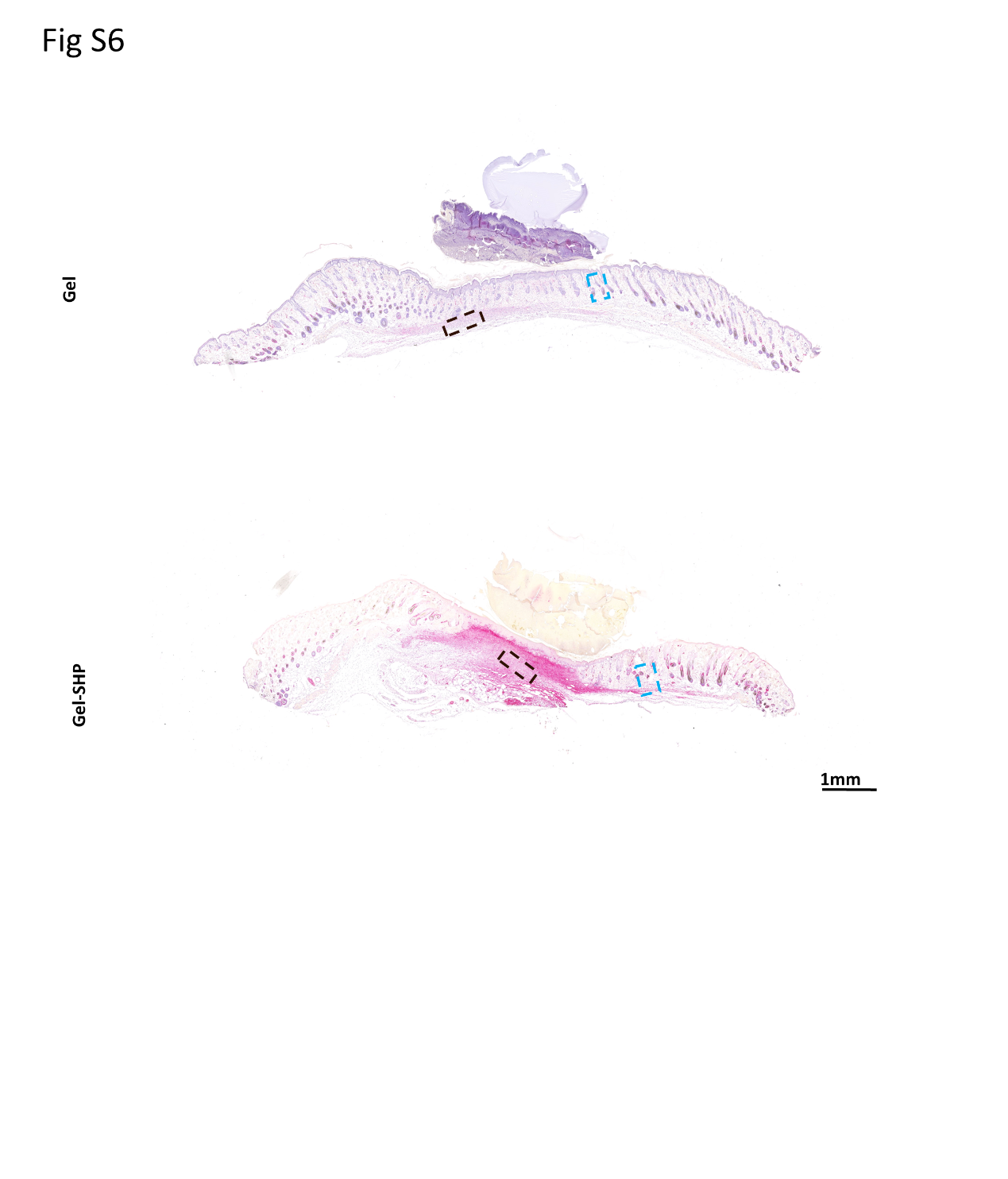


Figure S7 IHC stained sections after 7 days of Gel and Gel-SHP treatment of wounds (a-SMA, red).


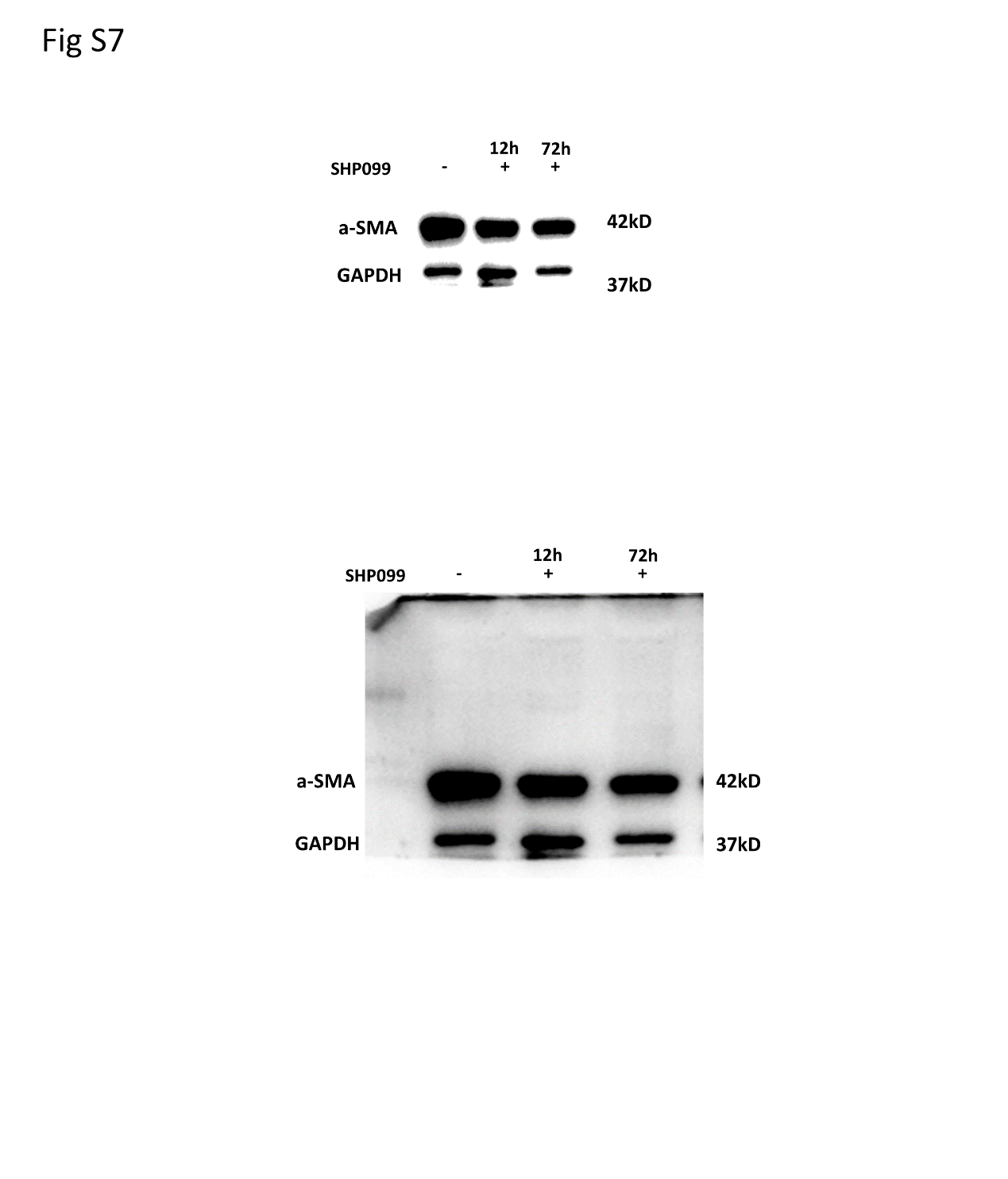


Figure S8 Western blot results of L929 showed that SHP099 did not significantly promote a-SMA expression in fibroblasts.


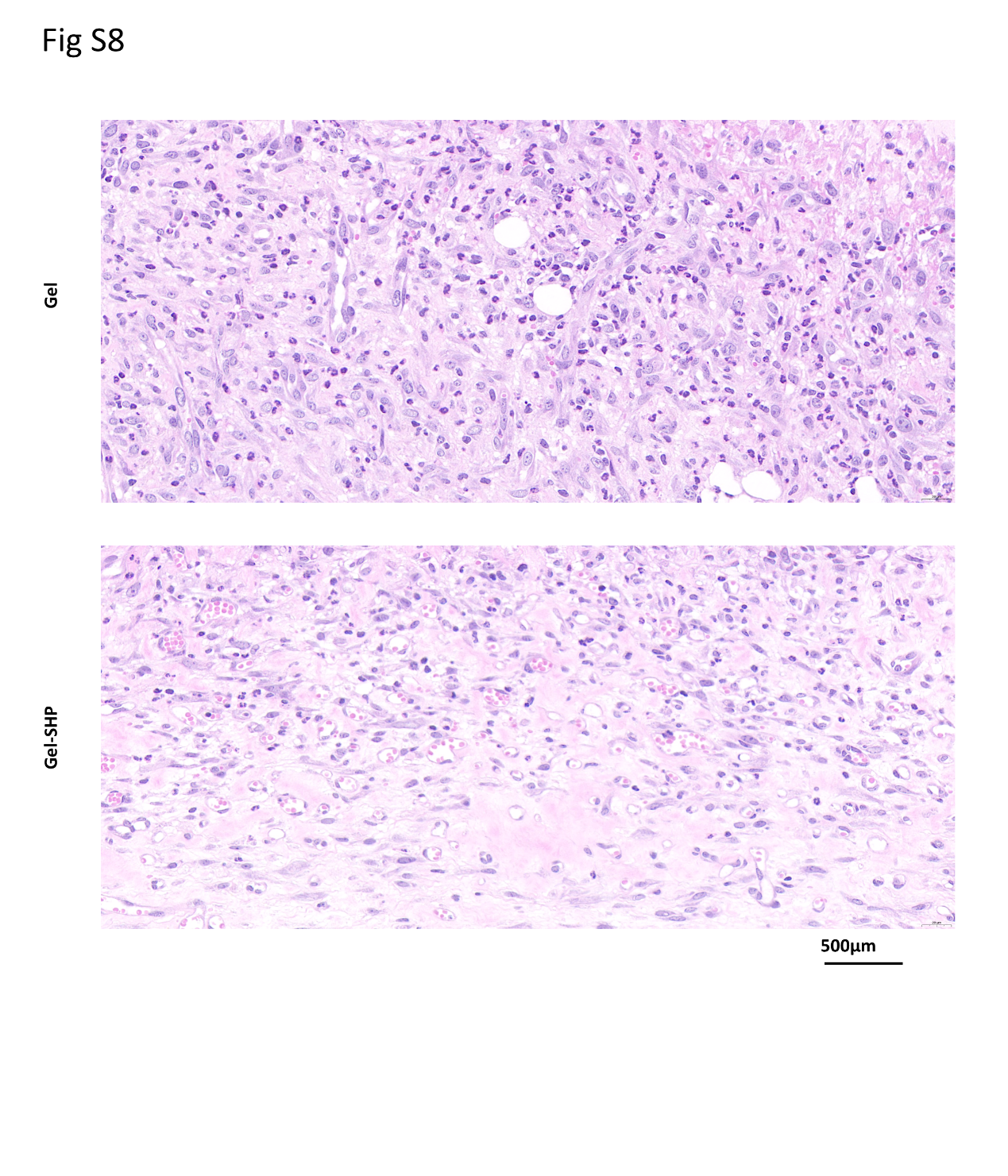


Figure S9 Sections stained with H&E after 7 days of Gel and Gel-SHP treatment of wounds (blue box in Figure S1)


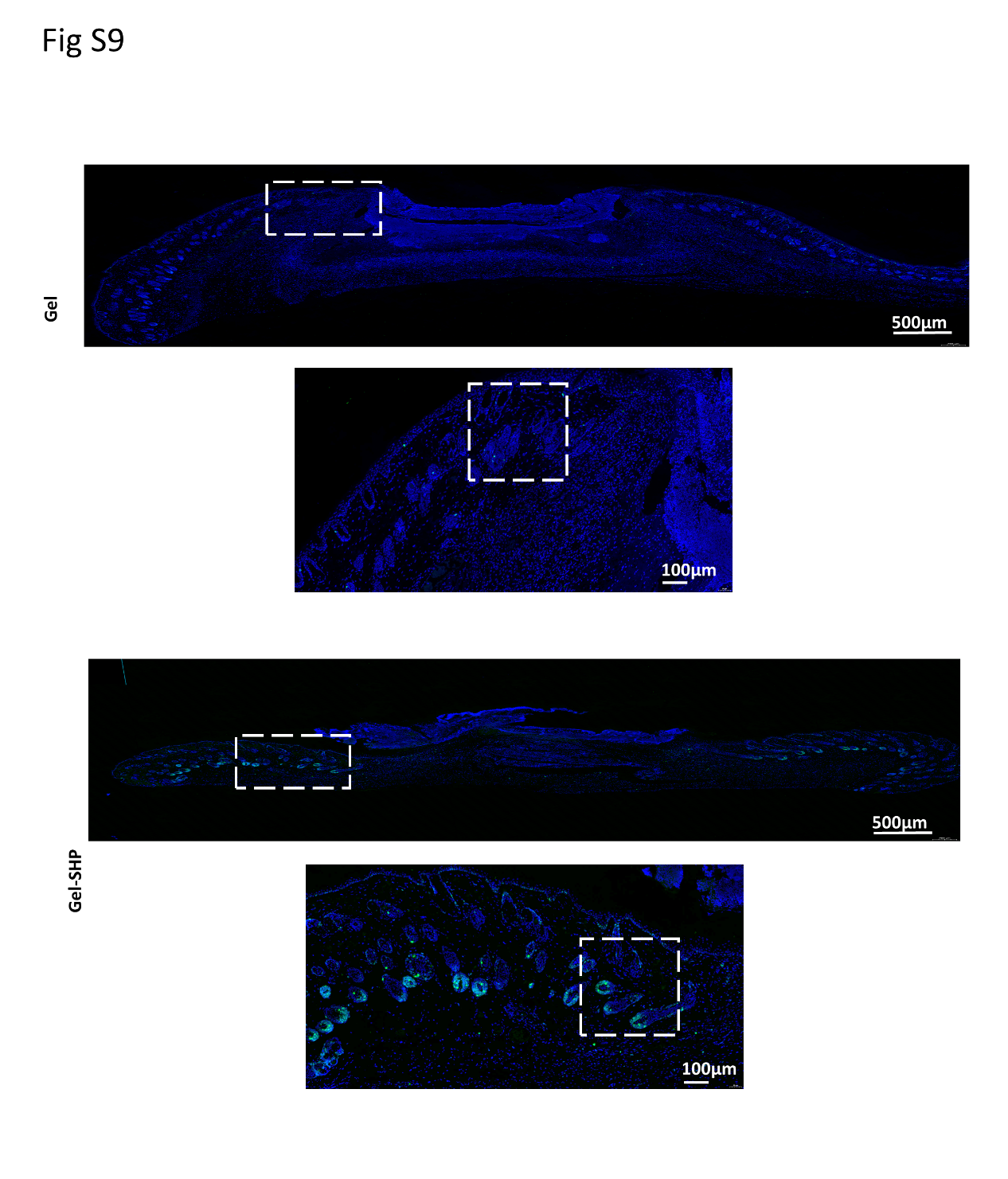


Figure S10 Immunofluorescence stained sections after 7 days of Gel and Gel-SHP treatment of wounds (Ki67, green; DAPI, blue).


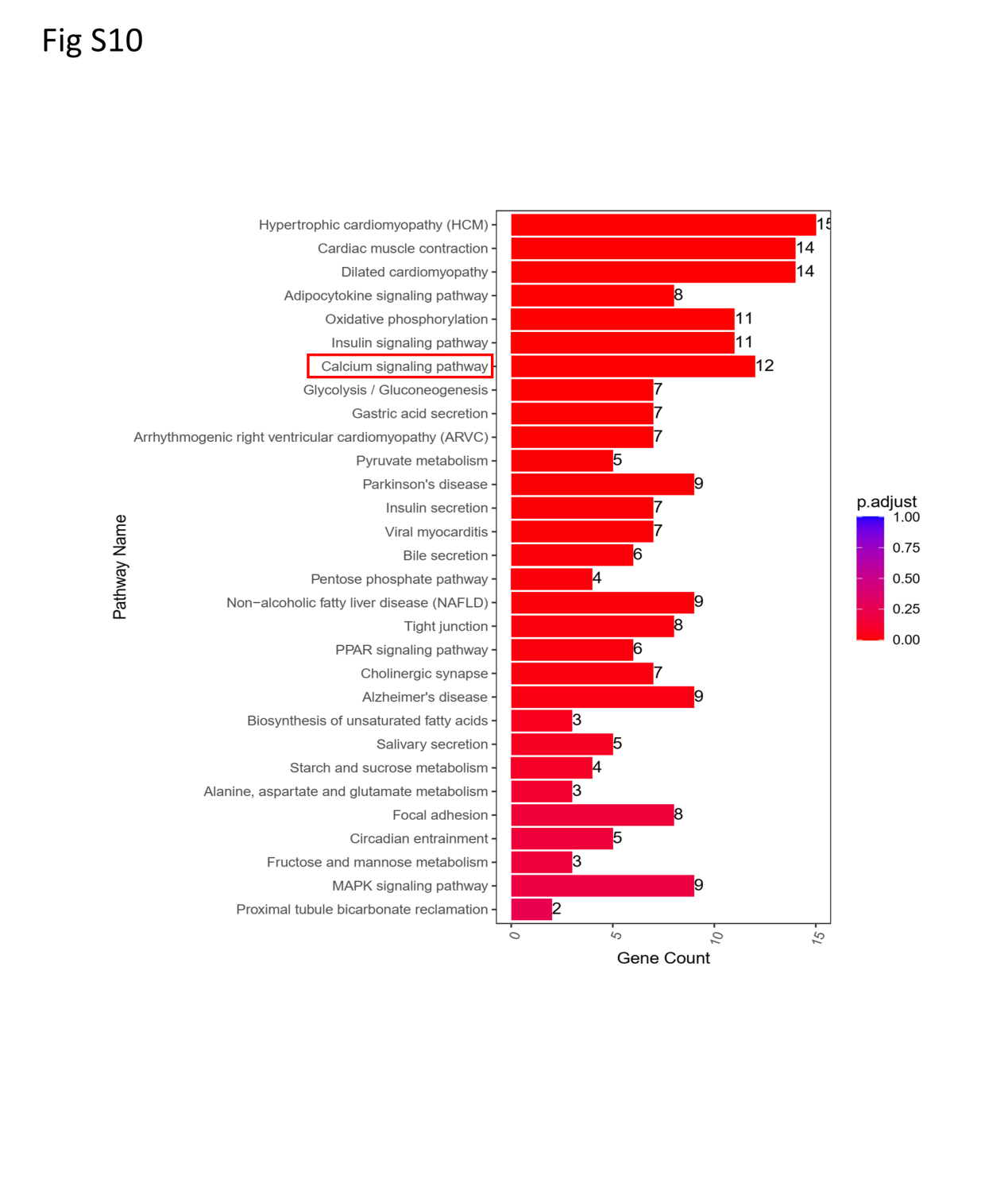


Figure S11 KEGG enrichment analysis of significant difference genes. Significant enrichment was defined as *p* value<0.05 (Gel-SHP group VS Gel group, Top 30)


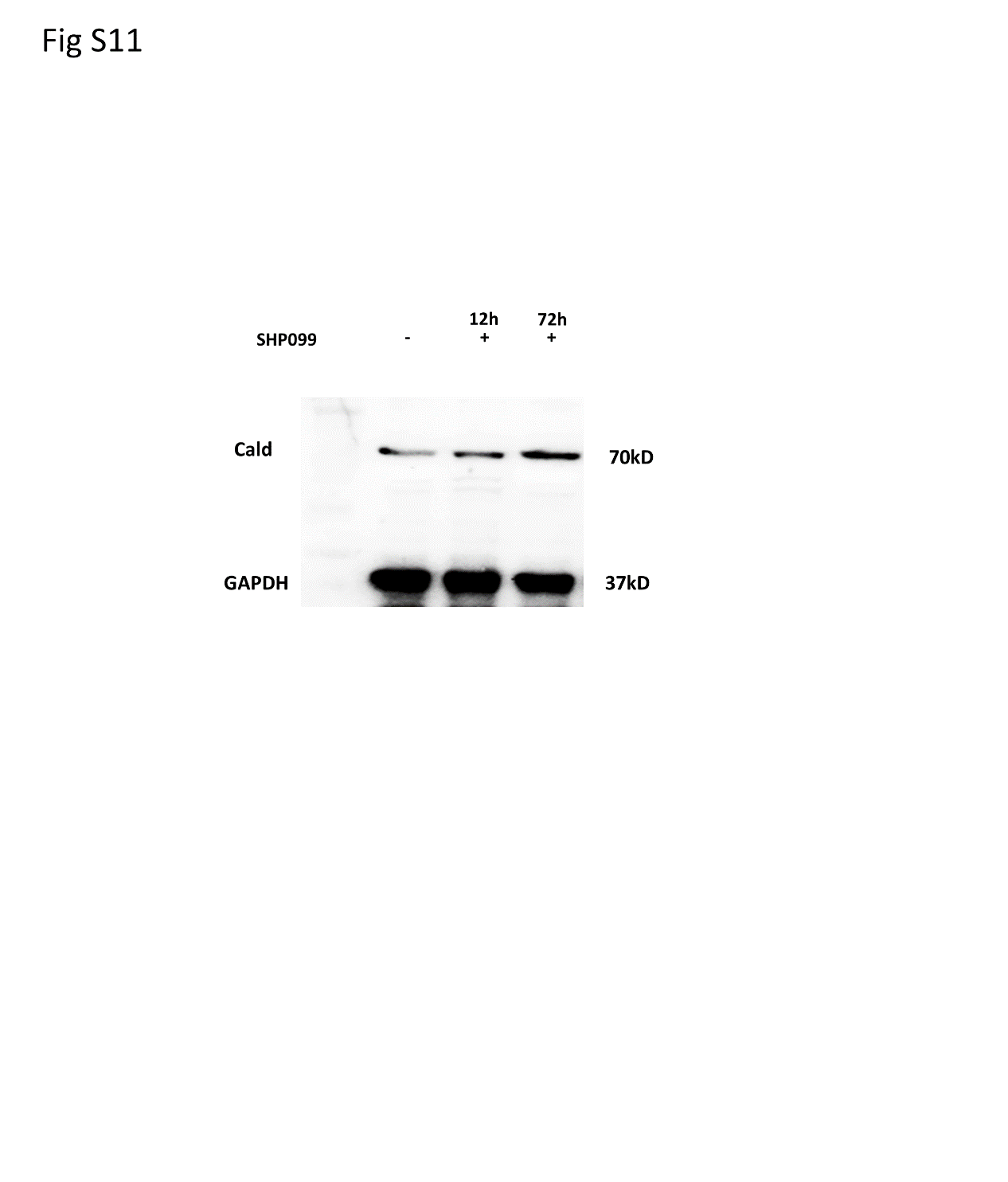


Figure S12 Western blot result of L929 showed that SHP099 promoted Cald1 expression in fibroblasts (raw data).


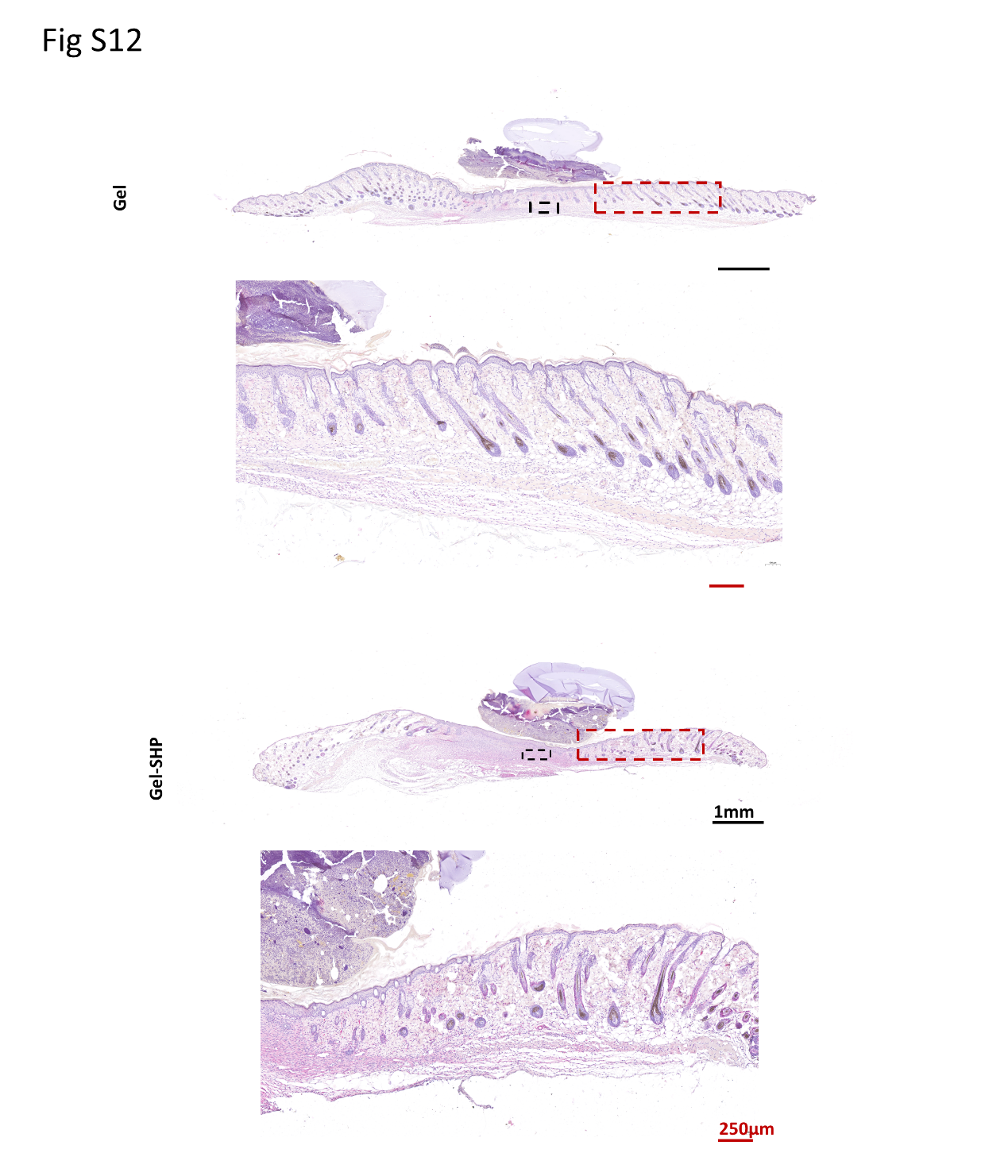


Figure S13 IHC stained sections after 7 days of Gel and Gel-SHP treatment of wounds (Cald1, red).
